# A phase II trial of stereotactic body radiotherapy with concurrent anti-PD1 treatment in metastatic melanoma: evaluation of clinical and immunologic response

**DOI:** 10.1186/s12967-017-1123-x

**Published:** 2017-01-31

**Authors:** Katrien De Wolf, Vibeke Kruse, Nora Sundahl, Mireille van Gele, Ines Chevolet, Reinhart Speeckaert, Lieve Brochez, Piet Ost

**Affiliations:** 1 Department of Radiation-Oncology, University Hospital Ghent, De pintelaan 185, 9000 Ghent, Belgium; 2 Department of Medical Oncology, University Hospital Ghent, Ghent, Belgium; 3 Department of Dermatology, University Hospital Ghent, Ghent, Belgium

**Keywords:** Cancer immunotherapy, Stereotactic body radiotherapy, Metastatic melanoma, Biomarkers, Immune monitoring

## Abstract

**Background:**

Antibodies blocking programmed cell death 1 (PD-1) have encouraging responses in patients with metastatic melanoma. Response to anti-PD-1 treatment requires pre-existing CD8+ T cells that are negatively regulated by PD-1-mediated adaptive immune resistance. Unfortunately, less than half of melanoma tumours have these characteristics. Combining anti-PD-1 treatment with other immunomodulating treatments to activate CD8+ T cells is therefore of vital importance to increase response rates and long-term survival benefit in melanoma patients. Both preclinical and retrospective clinical data support the hypothesis that radiotherapy increases the response rates to anti-PD-1 treatment by stimulating the accumulation and activation of CD8+ T cells in the tumour microenvironment. Combining radiotherapy with a PD-1 blocking antibody might therefore increase response rates and even induce long-term survival. The current phase II study will be testing these hypotheses and aims to improve local and distant tumour responses by exploiting the pro-immunogenic effects of radiotherapy in addition to anti-PD-1 treatment.

**Methods:**

The trial will be conducted in patients with metastatic melanoma. Nivolumab or pembrolizumab, both antibodies that target PD-1, will be administrated according to the recommended dosing schedule. Prior to the 2nd cycle, radiotherapy will be delivered in three fractions of 8 Gy to the largest FDG-avid metastatic lesion. The primary endpoint is the proportion of patients with a partial or complete response in non-irradiated metastases according to RECIST v1.1. Secondary endpoints include response rate according to immune related response criteria, metabolic response, local control and survival. To identify peripheral blood biomarkers, peripheral blood mononuclear cells and serum samples will be collected prospectively before, during and after treatment and subjected to flow cytometry and cytokine measurement.

**Discussion:**

The current phase II trial aims at exploring the suggested benefits of combining anti-PD-1 treatment and radiotherapy. The translational focus on immunologic markers might be suitable for predicting efficacy and monitoring the effect so to improve patient selection for future clinical applications.

*ClinicalTrials.gov Identifier* NCT02821182

## Background

Patients with metastatic melanoma had a median overall survival of only 6–9 months [1] until a breakthrough was achieved with novel immunomodulatory agents blocking specific immune checkpoints. Immune checkpoints, such as cytotoxic T lymphocyte-associated antigen 4 (CTLA-4), PD-1 and programmed cell death 1 ligand (PD-L1), are negative regulators of the immune system, that play critical roles in maintaining self-tolerance and modulating immune responses to protect normal tissue from immune collateral damage. Inhibition of these immune checkpoints by CTLA-4 blocking agents and anti PD-L1 antibodies is therefore able to reactivate T cells and restore anti-tumour immunity, resulting in impressive efficacy in patients with metastatic melanoma [2].

For these patients, antibodies targeting PD-1 have shown superior responses than those seen with CTLA-4 blocking agents, with response rates of 34% compared to 11% respectively [3]. Unfortunately, there still remain a substantial number of patients that do not obtain any clinical benefit. It is hypothesized that anti-tumour responses are limited by other immune inhibitory mechanisms present in the tumour microenvironment (TME). Patients who do not respond to PD-1 blocking agents typically have an immune suppressive TME hampering the activation of CD8+ cytotoxic T cells (CTLs). These patients may require the addition of other therapies that enhance anti-tumour immunity or circumvent immune inhibition. Potential candidates include other immunotherapies and radiotherapy.

Radiotherapy has important effects on the immune system and is able to shift the balance from tumour immune evasion towards tumour control [4]. Additionally, the best tumour control and tumour immunity are more likely to be achieved with high dose per fraction radiotherapy [5, 6]. By using stereotactic body radiotherapy (SBRT) we are able to safely deliver such high doses of radiation very precisely in a small number of fractions. Preclinical evidence clearly indicates that SBRT increases response rates and long-term survival of anti-PD-1 treatment by stimulating the accumulation and activation of CD8+ CTLs in the TME [7–10]. Considering the delicate interplay between both modalities, we have chosen to investigate a specific combination sequence in which 1 cycle of anti-PD-1 treatment will precede SBRT. This sequence allows the creation of a more immune permissive TME in which radiotherapy can induce the release of multiple tumour antigens causing the activation of tumour-specific CD8+ CTLs. The subsequent cycles of anti-PD-1 treatment may further stimulate the effector function of activated CD8+ CTLs by blocking the engagement of PD-1 with its ligand PD-L1.

The current phase II trial aims at exploring the suggested benefits of this combination. Considering the toxicity of immune checkpoint inhibitors and their high economical cost, it is of utmost importance to identify patients who are likely to respond to these treatments beforehand. Unfortunately, there are currently no validated markers available to pre-identify responders, and even the effects of checkpoint inhibitors on circulating immune cells remain unknown. We therefore will monitor circulating immune cells and cytokine levels, to identify the mechanism of response and resistance to therapy. We recently demonstrated that low levels of plasmacytoid dendritic cells and high expression of indoleamine 2,3-dioxygenase (IDO) in the peripheral blood of melanoma patients confer a negative prognosis, independent of disease stage. Systemic IDO, PD-L1 and CTLA-4 expression were also interconnected [11]. We will specifically focus on the relevance of these markers, as they could help elucidate the counter-regulatory mechanisms and provide predictive information.

## Methods

### Objectives

#### Primary objective

The primary objective of this trial is to determine the response rate as per RECIST v1.1 of the combination of anti-PD-1 with SBRT.

#### Secondary objectives

Secondary objectives are to determine the immune-related response rate of the combination treatment, metabolic response, local control, progression free survival (PFS) and toxicity. We will also analyse circulating immune cells, cytokine levels and markers in tumour tissue (if feasible) during treatment.

### Trial design

This phase II trial assesses the response rates of the anti-PD-1/SBRT combination. Nivolumab or pembrolizumab will be administrated according to the recommended dosing schedule. Prior to the 2nd cycle, SBRT will be delivered (24 Gy in three fractions) to the largest (max. diameter of 5 cm) fluorodeoxyglucose (FDG)-avid metastatic lesion. Table 1 shows a general scheme of the trial.

**Table 1:** General scheme of the trial

	Run-in period	Study period	Observation period	
SBRT		Three fractions of 8 Gy every other day		
Anti-PD-1 treatment	First cycle		Second cycle	Continuation

This trial uses a Simon two-stage optimal design, a design often used for phase II cancer clinical trials [12]. This design allows the assessment of the efficacy of a combination therapy in a relatively small number of patients.

### Outcome measures

#### Primary endpoint

- Objective response rate of the non-irradiated metastases as determined by the response evaluation criteria in solid tumours (RECIST) v1.1 [13]. Response rate will be defined as the percentage of subjects achieving either a complete or partial response at 6 weeks after the start of anti-PD-1 treatment. Further follow up imaging will be performed at the discretion of the treating physician.

#### Secondary endpoints

- Objective response rate of the non-irradiated metastases as determined by immune related response criteria (irRC) [14].
- Metabolic response of the irradiated and non-irradiated metastases based on the European Organization of Research and Treatment of Cancer (EORTC) 1999 criteria [15].
- Local control defined as the time between local irradiation and the moment the irradiated lesion shows an increase in size of ≥20%, according to the RECIST V1.1, confirmed by a consecutive assessment at least 4 weeks after first documentation.
- PFS: two types of PFS will be defined. One as the time from inclusion to documented disease progression according to RECIST v1.1 or death from any cause. The other as the time from inclusion to documented disease progression according to irRC or death from any cause.
- Acute and late toxicity due to the combination treatment will be scored using the Common terminology criteria for adverse events (CTCAE) version 4.0.

#### Exploratory endpoint

- Immunologic responses assessed using peripheral blood samples and analysed with fluorescence-activated cell sorting (FACS) phenotyping, functional testing, ultra-performance liquid chromatography (UPLC) and enzyme-linked immunosorbent assay (ELISA). If feasible, immunologic responses will also be assessed on tumour tissue using IHC.

### Study population

Patients with metastatic melanoma who did not receive previous immunotherapeutic treatment and have at least two measurable extracranial lesions.

#### Inclusion criteria

- Before patient registration, written informed consent must be given according to the International Council for Harmonisation of Technical Requirements for Pharmaceuticals for Human Use (ICH)/Good Clinical Practice (GCP), and national/local regulations.
- Histologically confirmed diagnosis of melanoma.
- Be able to provide tissue from an archival primary tissue sample or a newly obtained biopsy, for the evaluation of PD-L1 and other immune markers using immunohistochemistry (IHC).
- At least two extracranial measurable metastatic lesions per RECIST v1.1 and irRC. All radiological studies must be performed within 28 days prior to registration.
- Previous BRAF inhibitor when elevated lactate dehydrogenase (LDH) in patients with BRAF V600 mutations is allowed.
- Karnofsky Performance status >60.
- Age 18 years or older.
- Female participants of childbearing potential must be willing to use two methods of birth control or be surgically sterile, or abstain from heterosexual activity for the course of the study through 120 days after the last dose of study treatment.
- Female participants who are breastfeeding or plan to breastfeed should be instructed to discontinue nursing during treatment.
- Male participants must agree to use an adequate method of contraception starting with the first dose of study therapy through 120 days after the last dose of study treatment.
- Demonstrate adequate organ function defined as the following:
  - Serum aspartate and alanine aminotransferase (AST and ALT) levels ≤2.5× upper limit of normal (ULN) or ≤5× ULN in patients with liver metastases.
  - Serum total bilirubin ≤1.5× ULN or direct bilirubin ≤ULN for patients with total bilirubin level >1.5 ULN.
  - Serum creatinine ≤1.5× ULN.
  - Absolute neutrophil count ≥1000/mcL.
  - Platelets ≥75,000/mcL.
  - Hemoglobin ≥9 g/dL or ≥5.6 mmol/L.
- No history of active autoimmune disease requiring systemic treatment within the past 3 months or documented history of clinically severe autoimmune disease, or syndrome that requires systemic steroids or immunosuppressive agents.
- Subjects who have had another malignancy should be disease-free for 5 years, or should have a history of completely resected non-melanoma skin carcinoma or successfully treated in situ carcinoma.
- No evidence of interstitial lung disease.
- No uncontrolled central nervous metastases and/or carcinomatous meningitis.
- No prior radiotherapy interfering with the radiotherapy treatment in the study.
- No concomitant therapy with interleukin-2, interferon, other immunotherapy regimens, chemotherapy, immunosuppressive agent or chronic use of systemic corticosteroids.
- No active infection requiring systemic therapy.
- No known history of human immunodeficiency virus.
- No known active Hepatitis B or Hepatitis C infection.
- Subjects should not have received a live vaccine within 30 days prior to start of study treatment.
- Subjects without a mental condition rendering the patient unable to understand the nature, scope and possible consequences of the study.
- Subjects who are likely to comply with the protocol; i.e. no uncooperative attitude, no inability to return for follow-up visits, and likely to complete the study.

### Evaluation and randomization

Patients must be restaged within 4 weeks prior to randomization with an 18F-FDG Positron emission tomography with X-ray computed tomography (PET/CT).

### Intervention

#### SBRT

Prior to the 2nd cycle of treatment, SBRT will be delivered to the largest FDG-avid metastatic lesion (max. 5 cm diameter). A total dose of 24 Gy will be delivered in two fractions with image-guided treatment verification and fractions will be separated >48 h and <96 h.

All patients will receive a CT simulation in supine position with 2 mm CT slice thickness through the tumour site. The planning simulation should cover the target and all organs at risk. A typical scan length should extend at least 10 cm superior and inferior beyond the treatment field borders. Support devices to increase patient comfort will be chosen depending on the tumour localisation. The isocenter will be determined on the CT-simulator with marking of laser lines on the patient. Imaging data will be transferred to the treatment planning system. For all lesions, the Gross Target Volume (GTV) will be defined as all visible tumour by combining iconographic and metabolic information. No additional margin will be added for microscopic spread of disease. The GTV will be expanded with 2–5 mm to the Planning Target Volume (PTV) to account for organ motion and setup error. Margins depend on the site irradiated with 2 mm margins for bony lesions, 3 mm for nodes and 5 mm for other sites. The type of organ at risk delineated depends on the localization of the metastasis. A Planning Organ at Risk Volume (PRV) expansion of 2–5 mm will be added for organs at risk (OAR) and dose constraints apply to this PRV. It is strongly recommended that dose constraints not be exceeded. If a dose constraint cannot be achieved due to overlap of the target with an organ at risk or its PRV, the total dose can be lowered in order to meet the constraint. Treatment will be prescribed to the periphery of the target (80% of the dose) covering the 90% of the PTV. Dose constraints of organ at risks will be in accordance with the recommendations of the American Association of Physicist in Medicine (AAPM) task group 101 report. In case of violation of constraints to the organs at risk, the prescription will be adapted accordingly.

#### Systemic therapy

Anti-PD-1 treatment (nivolumab or pembrolizumab) will be administrated according to the recommended dosing schedule and continued until clinical progression. Patients may also discontinue protocol therapy when unacceptable toxicity is encountered. Administration of anti-PD-1 treatment should be withheld for a drug-related non-hematologic toxicity ≥grade 2 (excluding fatigue). The use of corticosteroids should be considered for management of immune-related adverse events. Once the patient has recovered to grade 0–1 consider increasing the dosing interval in subsequent cycles by 1 week. If the drug-related toxicity does not resolve to grade 0–1 within 12 weeks after onset of toxicity, discontinuation is recommended. Patients may also discontinue protocol in case of intercurrent illness, which would in the judgment of the investigator affect patient safety, the ability to deliver treatment or by request of the patient.

#### Evaluation of pre-treatment PD-L1 expression

A PD-L1 IHC assay using Merck mouse monoclonal antibody clone 22C3 will be performed on archival primary tissue sample or a newly obtained biopsy. Hematoxylin & eosin staining will be reviewed for confirmation of tumour presence.

#### Evaluation of the immunological response

The study requires blood samples (EDTA and serum) before start of anti-PD-1 treatment, before start of SBRT, 5–7 days after the last dose of SBRT and at week 6. The samples will be analysed with FACS phenotyping, functional testing and ELISA. The immune response will be analysed with a comprehensive immunophenotyping on peripheral blood. We will specifically look at the expression of immune suppressive markers CTLA-4, PD-L1 and IDO. For PD-L1 staining, we will use the mouse anti-human monoclonal antibody PD-L1 PE-Cy7. For intracellular staining, PBMCs will be fixed and permeabilized with fixation/permeabili-zation solution, and then stained with anti-human IDO PE and CTLA-4 APC. Tryptophan and kynurenine, a downstream metabolite of IDO, in patient’s sera will be quantified by UPLC-mass spectrometry. We will also look at absolute lymphocyte count, absolute neutrophil count/absolute lymphocyte count, serum tryptophan, C-reactive protein and cytokines, frequencies of Foxp3+ regulatory T cells, dendritic cell and myeloid derived suppressor cell subsets, next to functional analysis looking at shifts in Th1/Th2/Th17 polarization as a function of treatment [11, 16].

If feasible, tumour tissue will be analysed by IHC staining. Serial sections will be incubated with a monoclonal anti-FoxP3 and a monoclonal anti-IDO antibody for 1 h. For staining with CD3, CD8 and CD31 antibodies, an incubation time of 30 min will be used [17].

### Follow-up

Patients will be seen before the start of each treatment cycle during the whole course of anti-PD-1 therapy. At each visit, a history and physical examination will be conducted with recording of the toxicity. For response evaluation, a 18F-FDG PET/CT will be performed at week 6. For further follow up, CT thorax, abdomen and pelvis or 18F-FDG PET/CT will be performed at the treating physician’s discretion until disease progression or treatment discontinuation. Additional imaging or laboratory investigations should be carried out at the discretion of the treating physician, based on findings in the history or physical examination.

### Sample size

In the first stage, 20 patients will be accrued. If there are five or fewer responses, the alternative hypothesis will be rejected and the study will be stopped. If there are 13 or more responses, the null hypothesis will be rejected. Otherwise 20 additional patients will be accrued for a total of 40 patients (Table 2). Simon’s 2-stage Optimum design [18] will be used.

**Table 2:** Simon’s 2-stage optimum design

Optimal two stage design	Optimum design
First stage sample size (n1)	20
a1	6
b1	12
Maximum sample size (n)	40
b2	17

### Data analysis

- The goal of the proposed trial is to determine the efficacy of the proposed combination sequence of anti-PD-1 treatment and radiotherapy. The primary endpoint is the objective response rate as per RECIST v1.1. The null hypothesis that the true response rate is 0.34 [3] will be tested against a one-sided alternative. The null hypothesis will be rejected if 18 or more responses are observed in 40 patients.
- PFS is defined from the day of randomization until progression or last follow-up. Cases will be censored at last follow up visit if no progression was observed. Multivariate analysis will be performed according to the Cox-Regression method.
- For the evaluation of immunological markers over time, differences between groups will be tested by using the Friedman test. To compare proportions of categorical variables, the Pearson’s Chi^2^ test or Fisher’s Exact test will be used. To evaluate correlations, Spearman correlation coefficients will be calculated. All statistical analyses will be done on an ‘intention-to-treat’ basis and performed using SPSS 24.0 (SPSS Inc, Chicago, IL, USA), a P-value less than 0.05 will be considered statistically significant.

### Study approval

This trial is approved by the Ethics committee of the Ghent University Hospital (EC2016/0540) and is registered on clinicaltrials.gov (NCT 02821182).

## Discussion

Although current immunotherapeutic treatment options have led to an important breakthrough in patients with metastatic melanoma, they still fail to induce long-lasting clinical benefit in the majority of patients. We hypothesize that combining anti-PD-1 treatment with radiotherapy might result in improved clinical response rates and PFS compared to anti-PD-1 treatment in monotherapy. Both preclinical and retrospective clinical data support this hypothesis. The current study is an innovative translational phase II design translating preclinical data to the clinic. By using a Simon two-stage optimal design, the study will allow the assessment of the efficacy of a combination therapy in a relatively small number of patients before embarking on more expensive randomized trials. In addition, the translational focus on immunologic markers might be suitable for identifying mechanisms of response and resistance to therapy, resulting in predictors for efficacy and improved patient selection for future clinical applications.

## Abbreviations

AAPM: American Association of Physicist in Medicine
AST and ALT: aspartate and alanine aminotransferase
CTCAE: common terminology criteria for adverse events
CTLs: cytotoxic T cells
CTLA-4: cytotoxic T lymphocyte-associated antigen 4
ELISA: enzyme-linked immunosorbent assay
EORTC: European Organization of Research and Treatment of Cancer
FACS: fluorescence-activated cell sorting
FDG: fluorodeoxyglucose
GTV: Gross Target Volume
irRC: immune related response criteria
IHC: immunohistochemistry
IDO: indoleamine 2,3-dioxygenase
LDH: lactate dehydrogenase
OAR: organs at risk
PBMCs: peripheral blood mononuclear cells
PRV: Planning Organ at Risk Volume
PTV: Planning Target Volume
PET/CT: positron emission tomography with X-ray computed tomography
PD-L1: programmed cell death 1 ligand
PD-1: programmed cell death 1
PFS: progression free survival
RECIST: response evaluation criteria in solid tumours
SBRT: stereotactic body radiotherapy
TME: tumour microenvironment
UPLC: ultra-performance liquid chromatography
ULN: upper limit of normal

## References

[1] Kim C, Lee CW, Kovacic L (2010). Long-term survival in patients with metastatic melanoma treated with DTIC or temozolomide. Oncologist.

[2] Dummer R, Hauschild A, Lindenblatt N (2015). Cutaneous melanoma: ESMO Clinical Practice Guidelines for diagnosis, treatment and follow-up. Ann Oncol.

[3] Robert C, Schachter J, Long GV (2015). Pembrolizumab versus Ipilimumab in Advanced Melanoma. N Engl J Med.

[4] De Wolf K, Vermaelen K, De Meerleer G (2015). The potential of radiotherapy to enhance the efficacy of renal cell carcinoma therapy. Oncoimmunology.

[5] Schaue D, Ratikan JA, Iwamoto KS (2012). Maximizing tumor immunity with fractionated radiation. Int J Radiat Oncol Biol Phys.

[6] Dewan MZ, Galloway AE, Kawashima N (2009). Fractionated but not single-dose radiotherapy induces an immune-mediated abscopal effect when combined with anti-CTLA-4 antibody. Clin Cancer Res.

[7] Deng L, Liang H, Burnette B (2014). Irradiation and anti-PD-L1 treatment synergistically promote antitumor immunity in mice. J Clin Investig.

[8] Sharabi AB, Nirschl CJ, Kochel CM (2015). Stereotactic radiation therapy augments antigen-specific PD-1-mediated antitumor immune responses via cross-presentation of tumor antigen. Cancer Immunol Res.

[9] Zeng J, See AP, Phallen J (2013). Anti-PD-1 blockade and stereotactic radiation produce long-term survival in mice with intracranial gliomas. Int J Radiat Oncol Biol Phys.

[10] Gerber SA, Lim JY, Connolly KA (2014). Radio-responsive tumors exhibit greater intratumoral immune activity than nonresponsive tumors. Int J Cancer.

[11] Chevolet I, Speeckaert R, Schreuer M (2015). Characterization of the in vivo immune network of IDO, tryptophan metabolism, PD-L1, and CTLA-4 in circulating immune cells in melanoma. Oncoimmunology.

[12] Mander AP, Thompson SG (2010). Two-stage designs optimal under the alternative hypothesis for phase II cancer clinical trials. Contemp Clin Trials.

[13] Eisenhauer EA, Therasse P, Bogaerts J (2009). New response evaluation criteria in solid tumours: revised RECIST guideline (version 1.1). Eur J Cancer.

[14] Wolchok JD, Hoos A, O’Day S (2009). Guidelines for the evaluation of immune therapy activity in solid tumors: immune-related response criteria. Clin Cancer Res.

[15] Young H, Baum R, Cremerius U (1999). Measurement of clinical and subclinical tumour response using [18F]-fluorodeoxyglucose and positron emission tomography: review and 1999 EORTC recommendations. European Organization for Research and Treatment of Cancer (EORTC) PET Study Group. Eur J Cancer.

[16] Chevolet I, Speeckaert R, Schreuer M (2015). Clinical significance of plasmacytoid dendritic cells and myeloid-derived suppressor cells in melanoma. J Transl Med.

[17] Chevolet I, Speeckaert R, Haspeslagh M (2014). Peritumoral indoleamine 2,3-dioxygenase expression in melanoma: an early marker of resistance to immune control?. Br J Dermatol.

[18] Simon R (1989). Optimal two-stage designs for phase II clinical trials. Control Clin Trials.

